# Pregnant Women’s Attitudes Toward and Experiences With a Tablet Intervention to Promote Safety Behaviors in a Randomized Controlled Trial: Qualitative Study

**DOI:** 10.2196/28680

**Published:** 2021-07-20

**Authors:** Bente Walter, Hege Indreboe, Mirjam Lukasse, Lena Henriksen, Lisa Garnweidner-Holme

**Affiliations:** 1 Department of Nursing and Health Promotion Oslo Metropolitan University Oslo Norway; 2 Faculty of Health and Social Sciences University of South-Eastern Norway Vestfold Norway

**Keywords:** intimate partner violence, eHealth, pregnancy, antenatal care, safety behaviors, tablet intervention

## Abstract

**Background:**

Intimate partner violence (IPV) is recognized as a global health problem. Women with low education and limited resources are more vulnerable, as are immigrant women. There is a lack of evidence on how health care professionals should communicate about and intervene against IPV during pregnancy. Earlier research has shown that when women manage digital questionnaires, they are more likely to disclose IPV. However, little is known about how women experience eHealth interventions with safety behaviors to prevent IPV.

**Objective:**

The aim of this study was to explore pregnant women’s attitudes toward and experiences with a tablet intervention to promote safety behaviors in a randomized controlled trial (RCT) in antenatal care.

**Methods:**

Individual semistructured interviews were conducted with 10 women who participated in the Safe Pregnancy Study. The Safe Pregnancy Study was a randomized controlled trial (RCT) using a tablet intervention containing IPV questions and a film to promote safety behaviors. Six women from the intervention group and four women from the control group were recruited. The content was available in Norwegian, Somali, and Urdu. Five of the women participating in the interviews spoke Norwegian at home and five spoke another language. The majority of the women who did not speak Norwegian at home perceived themselves as relatively well integrated. The interviews were conducted at different maternal and child health centers (MCHCs) in Norway between March 2020 and June 2020. The analysis was guided by thematic analysis.

**Results:**

Women who participated in the tablet intervention appreciated being asked questions about IPV on a tablet. However, it was important to supplement the tablet intervention with face-to-face communication with a midwife. The MCHC was regarded as a suitable place to answer questions and watch a film about safety behaviors. Women suggested making the tablet intervention available in other settings where women meet health care professionals. Some women expressed uncertainty about their anonymity regarding their answers in the questionnaire. We found no real differences between ethnic Norwegian and immigrant women’s attitudes toward and experiences with the tablet intervention.

**Conclusions:**

Questions about IPV and a film about safety behaviors on a tablet, as a supplement to face-to-face communication, might initiate and facilitate communication about IPV in antenatal care. Uncertainty regarding anonymity has to be addressed when questions about IPV are being asked on a tablet.

**Trial Registration:**

ClinicalTrials.gov NCT03397277; https://clinicaltrials.gov/ct2/show/NCT03397277

## Introduction

### Background

Intimate partner violence (IPV) is recognized as a global health problem [1]. According to the World Health Organization, IPV may include physical aggression, sexual coercion, psychological abuse, or controlling behaviors by current or former partners [1]. IPV can occur among people of any gender, identity, or sexual orientation and does not require sexual intimacy [2]. Almost one-third of women worldwide have been exposed to physical and/or sexual violence by an intimate partner throughout their life [2]. A meta-analysis of IPV during pregnancy, which included 92 studies from 23 countries, reported a prevalence of physical abuse of 13.8%, sexual abuse of 8.4%, and emotional abuse of 28.4% [3]. In Norway, the prevalence of IPV was reported to range from 1% to 5% in different studies [4,5]. IPV has been associated with multiple adverse physical and mental health conditions among women of all backgrounds [6]. Pregnancy does not protect against IPV [7,8]; it can be a motivator to stay in the relationship or a driving force to break out of a violent relationship [7]. IPV prior to pregnancy, during pregnancy, or in the neonatal period is associated with depression, unwanted pregnancy, miscarriage, stillbirth, premature birth, or intrauterine growth restriction [4,9-11]. IPV occurs in all social strata. Women with low education and limited resources are more vulnerable, as are immigrant women [12].

The Norwegian guidelines for antenatal care strongly recommend that all women should be asked about current and/or past experiences of IPV [13]. There is a lack of evidence on how health care professionals should communicate about and intervene against IPV during pregnancy [8,14]. Women exposed to IPV report weaknesses in conversations about violence with health care professionals, which could reduce the possibility to disclose their experiences [7,15,16]. Health care providers report challenges in conversations about IPV due to personal barriers and lack of knowledge about how to intervene [17-20]. Studies have examined eHealth interventions in IPV screening in different settings and patient populations. They show that women are more likely to disclose IPV when they use digital tools in violence screening [17,21].

Qualitative research alongside randomized controlled trials (RCTs) can improve the understanding and effects of complex health care interventions [22]. Other studies have conducted qualitative research as part of the process evaluation phase of RCTs concerning IPV [23-25]. This research provided insights into the complexity of women’s help-seeking behaviors and the factors that influence the experience of different kinds of support [24,26]. The aim of this study was to explore pregnant women’s attitudes and experiences with a tablet intervention to promote safety behaviors as part of an RCT in antenatal care.

### The Safe Pregnancy Study

The Safe Pregnancy Study is an RCT to test the effectiveness of a tablet-based, culturally adapted intervention in antenatal care aiming to promote safety behaviors and prevent IPV among Norwegian, Pakistani, and Somali women [4]. The RCT was performed in a routine antenatal care setting at 19 maternal and child health centers (MCHCs) in Norway. The recruitment took place between January 2018 and June 2019 [4], and 317 women participated in the study.

Upon giving consent to participate in the study, all participants were asked to answer a baseline questionnaire on a tablet. A modified version of the Abused Assessment Screen (AAS) [27,28] was part of the baseline questionnaire. Exposure to violence was determined by a positive response to at least one of the five questions in the AAS. Women who screened positive in the AAS were included in the RCT and received detailed questions on IPV and their experiences with actions to make themselves safer. Subsequently, they were randomized to an intervention group or a control group. Women in the intervention group watched a film about the nature of IPV and behaviors that can increase safety [4]. Women in the control group watched a film with general information about health during pregnancy, including information about where to find help if experiencing violence. User involvement was highly prioritized, and women from Norway, Pakistan, and Somalia participated in the development process to culturally adapt the intervention film [4]. The intervention film and the control film lasted for approximately 7 minutes.

The questionnaire included items about pregnancy, health, quality of life, IPV, education, language, income, and personal perception of integration. Women who screened positive in the AAS were asked to complete the Composite Abuse Scale R-SF (CAS), an instrument containing 15 questions about physical, psychological, and sexual violence [29,30], and the Safety Behavior Questionnaire [31,32].

In this study, we defined tablet intervention as the use of an electronic device to pose detailed questions on IPV and safety behaviors and to show a film. However, the film in the RCT differed for the control and interventions groups, as described above. Thus, all participated in the tablet intervention, but only the intervention group, viewed the film on IPV and safety behaviors.

## Methods

### Interviews

Semistructured individual interviews with 10 women who participated in the Safe Pregnancy RCT were conducted by BW and HI (master’s students in midwifery). LGH (qualitative researcher) participated in the pilot interview. Interviews were conducted in Norwegian between March 2020 and June 2020. Nine interviews were performed in an office or meeting room at a MCHC, and one interview took place in a private room in a library. The interviews lasted from 21 minutes 23 seconds to 53 minutes 43 seconds. The interviews followed a semistructured interview guide developed by the interdisciplinary research group in the Safe Pregnancy Study. The interview guide had the following three themes: (1) background for participation, (2) experience with the questionnaire, and (3) experience with the film about safety behaviors. A pilot interview was conducted to test the interview guide, and this interview was included in the analysis. Findings from the pilot interview were that the women had poor recollection of the questionnaire and film. The interview guide was therefore modified before the next interview took place. The themes in the interview guide remained unchanged, but the questions were changed to a descriptive form with short and simple questions to obtain spontaneous descriptions. The interdisciplinary research group decided that the women should have the opportunity to see the questions and the film again during the interview (Multimedia Appendix 1).

### Recruitment

Women were recruited through phone calls by BW and HI. Inclusion criteria for individual interviews were women who spoke adequate Norwegian and had participated in the Safe Pregnancy RCT Study in 2019. BW and HI received lists of participants with phone numbers from the Safe Pregnancy Study register. The first five women who agreed to participate were chosen from a list with participants from both the control and intervention groups. All received detailed questions about the experience of violence and use of safety behaviors, and only the intervention group viewed the film with information about the nature of violence and behaviors to increase safety. After conducting and transcribing the interviews from the control group, BW, HI, and LGH found that little new nuanced information was added to the data set. Hence, the last five women were chosen from a register with participants in the intervention group only. Recruitment was carried out until a rich set of data was obtained. The Regional Committee for Medical and Health Research Ethics approved the study (reference number 2017/358). The participants provided their written consent.

### Analysis

The analysis was guided by thematic analysis, as described by Braun and Clarke [33], and included the following steps: (1) Familiarizing with the data by repeated reading of each informant’s transcript; (2) Generating initial codes that were relevant to the research question across the entire data set; (3) Organizing the codes into subthemes; (4) Arranging the subthemes into overreaching themes; and (5) Defining and naming the themes.

Interviews were audiotaped and transcribed by BW and HI. BW and HI compared a random part (6 minutes) of each other’s transcripts with the audiotapes to ensure accuracy of the transcription process. LGH, LH, and ML read all the transcripts during the interview process. BW and HI performed the analysis guided by LGH. BW and HI together coded each transcript. The process of generating initial codes and findings of potential themes and subthemes was discussed with LGH. ML and LH participated in the discussion of defining and naming the themes to strengthen the reliability of the findings. A qualitative software program (Nvivo 12 Pro) was used to identify codes and systematize subthemes.

## Results

### Characteristics of the Women

Six women from the intervention group and four women from the control group were recruited. Five of the women spoke Norwegian at home and five spoke another language. The majority of the women who did not speak Norwegian at home perceived themselves as relatively well integrated. Time from participation in the tablet intervention to the interview varied from 11 to 25 months (Multimedia Appendix 2).

The analysis resulted in the following themes representing the women’s attitudes and experiences with a tablet intervention to promote safety behaviors: (1) Positive attitudes toward and experiences with questions asked about IPV via a tablet shown at the MCHC; (2) Negative attitudes toward and experiences with the film about IPV via a tablet shown at the MCHC; (3) Positive attitudes toward and experiences with the film about IPV on a tablet shown at the MCHC; (4) Motivation to participate in the Safe Pregnancy Study; and (5) Women’s suggestions for improvements to the tablet intervention (Table 1).

**Table 1:** Themes and subthemes.

Theme and subtheme			Identified in the control (C) and/or intervention group (I)
**Positive attitudes toward and experiences with questions about IPV^a^ via a tablet shown at the MCHC^b^**			
	Remembered the questions related to IPV because they were unexpected	I	
	The MCHC was a safe place to be asked questions related to IPV on a tablet	C and I	
	Using a tablet enabled honest answers in a nonconfrontational setting without being disrupted	C and I	
	Provided an opportunity for a conversation with the midwife	C and I	
	Easy to answer when you can choose your own mother tongue	C and I	
	Reflected about their own life situation	C and I	
**Negative attitudes toward and experiences with questions about IPV via a tablet shown at the MCHC**			
	The questions gave the feeling of being interrogated	C	
	Have thought about the questions since	I	
**Positive attitudes toward and experiences with the film about IPV via a tablet shown at the MCHC**			
	The MCHC was a safe environment to watch a film about IPV	I	
	The film gave a feeling of not being alone in experiencing IPV	I	
	Information given in the film can be of importance for women with different cultural backgrounds	I	
	Made them aware of different types of IPV and the situation for victims of IPV	I	
**Motivation to participate in the Safe Pregnancy Study**			
	Wanted to contribute with experience and knowledge that could improve the service at the MCHC	C and I	
	Usually participated in research projects	C and I	
	IPV was a topic of importance	C and I	
	Wanted to be integrated	I	
**Women’s suggestions to improve the tablet intervention**			
	Women who claim they are living with IPV should be invited to a second consultation with the midwife	I	
	Believed that women who are exposed to IPV should be given the chance to consent for receiving help	I	
	Lacked contact details for the support services when they answered questions about IPV	I	
	Of the opinion that the tablet intervention is appropriate in other settings	I	
	It should be clearer in the film that abused women are not alone in their experience and that they can feel safe contacting supportive services	I	

^a^IPV: intimate partner violence.

^b^MCHC: maternal and child health center.

### Attitudes Toward and Experiences With Questions Asked About IPV Via a Tablet Shown at the MCHC

Most of the women remembered the questions about IPV from the tablet intervention. A woman from the intervention group said that she remembered the questions because she was surprised being asked about IPV.

Obviously…I remember the questions I was shocked by.

Informant 8

Women who did not remember the questions said that time from participation in the tablet intervention could have contributed to the lack of memory. They said that they received a lot of information during pregnancy, and there was a possibility that they mixed it up.

The women considered the MCHC as a safe and suitable place to be asked about IPV on a tablet, whether or not they had been exposed to IPV. They appreciated the opportunity to answer the questions alone, without a partner, as described by a woman in the control group as follows:

I think it’s a very smart place to get it. After all, this is a place where you can go and talk about your problems related to the pregnancy.

Informant 1

The women explained that questions about IPV on a tablet gave them the opportunity to first consider the questions undisrupted before answering. It felt safe, and it was positive that visitors and employees at the MCHC could not observe their body language while answering the questions. Most of the women believed it was easier to answer honestly on a tablet than face to face. One woman expressed how she found it as follows:

It is much easier when you’re able to think…have that extra time to read for yourself…and consider yes because I sometimes wondered was it a common quarrel…it is a very good thing that you have time…[to] make some reflections and considerations.

Informant 8

Some women felt that questions about IPV on a tablet opened for a conversation with the midwife. Being asked on a tablet led to a feeling that the topic was not unknown for the midwife, and it felt safe to talk with her. A woman who had been exposed to IPV and was an immigrant felt relieved when she was asked about IPV and said that it felt good. She reflected upon this as follows:

It was an opportunity for me, if I wanted to tell something to the midwife afterwards or the next visit…I felt confident that I could talk to her because it was not unknown to her.

Informant 6

Most of the women, independent of which group they belonged to, did not have issues answering the questions about IPV because they experienced them as concrete and distinct. One bilingual Norwegian woman valued the opportunity to answer the questions in her mother tongue.

Many women believed that it was important to be asked about IPV because it was a common reality of everyday life. Most experienced the questions as being relevant, although one woman from the control group had a different opinion as follows:

To answer such questions about violence, you go through your own thoughts really thoroughly, you think what do you want to answer? What do you not want to answer? Will it be saved? Is it an interrogation? You become a little skeptical.

Informant 5

The questions about IPV made an impression on some of the women. One described that it was painful to be reminded about her own experiences with IPV. Another woman who had been exposed to IPV commented that she had thought about the questions after participating in the tablet intervention.

I actually have, it may sound very stupid, but I have thought about it a lot…I was a bit thoughtful, by the questions, absolutely. After the last child was born, I have thought a lot more about things, probably…because of this questionnaire and the video and that kind of thing.

Informant 9

Some of the women said that the questions made them reflect about their own life situation. Most women mentioned they were grateful of being in a good relationship with their current partner. A woman from the control group said the following:

It is something about the deeper questions that you may not usually think about, how satisfied you are with things. But, it felt a bit nice to reflect over it, and that, yes, I'm pretty good.

Informant 4

One woman expressed her gratitude to her husband as follows:

I realized that it is a reality in many families, but I was a little proud of my husband, who is very nice and loving to me.

Informant 1

### Attitudes Toward and Experiences With the Film About IPV on a Tablet Shown at the MCHC

Most women did not remember anything from the film, and all of them chose to watch it during the interview. They perceived the MCHC as a safe place to watch a film about IPV, noting that not every woman was safe in her own home. A woman from the intervention group described the film as follows:

It can really give a feeling that there is someone who cares here, and maybe there are some ways out…the film gives concrete advice…I got the impression that it is perhaps easy to do the things like contacting the police or women’s shelter or just talk to the midwife…immediately when you are at the MCHC.

Informant 8

A woman with an immigrant background expressed that the film could be of importance for women who lacked knowledge about their civil rights. She felt that immigrant women needed to know where they could receive help if they were a victim of IPV. After watching the film again during the interview, many participants understood that IPV was a reality for some women. They thought that the film could help women who had experienced IPV feel that they were not alone. Some women stated that the film made them aware of different types of IPV and that it was important to receive this information. One participant who had experienced IPV was of the opinion that physical violence was more obvious and therefore easier to detect than psychological violence.

When it is physical violence, it is quite clear to a woman it is violence…it is important that they mention there is also violence when they control you…I think it has a lot of information and describes very well if you have doubts about whether you are in a violent relationship or not, it is very well described.

Informant 6

Some women thought that the film was useful because they learned how victims of IPV could experience their lives, and it could contribute to care for these women.

It is also useful for me to be aware that someone can experience it, and maybe when I talk to other pregnant women…be concerned that someone may experience it…if I see it, respond to it.

Informant 8

### Motivation to Participate in the Safe Pregnancy Study

The women had different reasons for participating. Some wished to participate because they wanted to influence and contribute to improvement of the service at the MCHC. Others were motivated to participate if it would help to improve the intervention. Informant 6 expressed her motivation as follows:

I decided it was very important for me to participate, but also give feedback to develop the program.

Informant 6

Like others, she also thought that the topic was important.

For me it was very important, it is a reason why I am here today, because I found it especially important.

Informant 6

Many stated that they participated because they usually participate in research projects. They believed that someone had to participate to make research possible, as described by a woman who was in the control group as follows:

I usually participate in most research projects I am asked to participate in…I don't mind…it is important to receive information.

Informant 3

Informant 10 thought that participating in the tablet intervention and the interview could help her to become integrated into Norwegian society.

Because I also need to talk…because I came here to learn something new…and at least integrate into society, which I have not before.

Informant 10

### Women’s Suggestions to Improve the Tablet Intervention

Women with an immigrant background thought that it was important to make the film available elsewhere, not only at the MCHC. They suggested that general practitioners’ offices or Norwegian language courses could be good locations. They thought that by doing this, women could receive the information at an earlier stage.

Informant 6 suggested that there should be an opportunity for women to consent in the questionnaire if they wanted help with IPV. She reported that women who stated they were exposed to IPV may want help and therefore should be invited to a new consultation with a midwife.

Informant 9 had the opinion that the tablet intervention was not good enough alone and should be supplemented with support from health care professionals.

It is a bit incomplete, in a way…if you are going to start with it, then you have to get a little closer to the person in an individual way.

Informant 9

It was suggested that there should be contact information for support services when women answered the questionnaire. Informant 6 felt it was important that information in the film about support services was given in a way that assures women are safe if they wish to contact them. She also thought that the film should contain more information on IPV being a common problem and women being not alone in experiencing IPV.

Many families experience problems with violence, you are not alone and maybe something that makes the woman trust a crisis center or makes it feel close, because it isn’t easy to just call a number, I need help. That is a very, very, very difficult thing to do.

Informant 6

## Discussion

### Principal Findings

This study showed that women who participated in the tablet intervention had positive attitudes and experiences to answer questions and see a film about IPV on a tablet. The MCHC was regarded as a suitable place for questions and a film on IPV, particularly when staff followed up on it. The majority of the women in this study found the questions on the tablet intervention easy to answer. However, it was important to supplement the questions and film with face-to-face communication with the midwife. Furthermore, they suggested making the tablet intervention available in other settings, such as Norwegian language courses and general practitioners’ offices. Women’s negative attitudes were mainly related to uncertainty about their anonymity regarding the answers they gave in the questionnaire.

### Comparison With Previous Work

Other studies have identified women’s positive attitudes toward and experiences with eHealth tools to communicate about IPV [23-25]. Women in our study said that questions and a film about IPV on a tablet provided an opportunity for a conversation with the midwife because they felt that the topic was known to them. Bacchus et al [25] conducted semistructured interviews with participants in an RCT with a computer tablet intervention for IPV. They found that women regarded a computer tablet intervention for IPV screening during perinatal home visits as an opportunity to talk to health professionals, and it gave women a feeling of being cared for. This is supported by Chang et al [23], who performed a qualitative study to compare in-person and computerized screening for IPV. They found that electronic questions about IPV prepared women to talk about their experiences with their health care provider.

Women in our study acknowledged the opportunity to answer the questions about IPV on a tablet undisturbed. Other studies have found that questions about IPV on a tablet are regarded as a safe and confidential way for abused women to disclose their experiences of IPV [18,19,23,25]. Women in this study expressed that they were able to reflect on the questions while answering alone, which in turn probably enhanced their level of honesty. Tarzia et al [24] did a qualitative study as part of an evaluation process of two Australian RCTs about safety for women experiencing IPV. They found that women exposed to IPV appreciated answering questions about violence in private and reflected about their life situation via an interactive online intervention. Previous studies have found that women who are exposed to IPV express challenges in disclosing this in face-to-face situations because they are worried about confidentiality and judgmental responses [7,16,19,23,34]. Interventions delivered via the internet have been rated as a preferred alternative method to face-to-face support for women experiencing IPV [24]. This might have the potential to overcome some barriers associated with accessing face-to-face screening [24]. Self-administrated questionnaires for IPV, including computer-based assessment tools, may thus be more effective than face-to-face questioning [17].

However, women who had positive attitudes toward and experiences with the tablet intervention in our study expressed that the tablet intervention should be supplemented with face-to-face support from health care professionals. A trusting relationship in a face-to-face intervention can create a safe place to talk, facilitate disclosure, and meet individual needs [24]. Bacchus et al [25] also found that a trusting relationship with a provider as a supplement to eHealth intervention was important to achieve disclosure of IPV. Chang et al [23] found that in-person screening provided for tailored questioning and more emotional connection with the provider and suggested that both in-person intervention and computerized screening were good methods for women to disclose IPV.

In line with this study, an earlier Norwegian study showed that women regarded the MCHC as an appropriate place to receive information about IPV [16]. Antenatal care was regarded as a suitable place to talk about IPV because pregnant women make repeated visits during their pregnancy [35,36]. The visits at an antenatal clinic can help create a trusting relationship between the pregnant woman and the health care provider [35]*.* Women in this study suggested that places other than MCHCs, such as general practitioners’ offices and Norwegian language centers, could also be appropriate places to introduce the tablet intervention. Other settings for IPV interventions have been examined. Bacchus et al [25] found that an eHealth tool for IPV could successfully be used in perinatal home visits. Bacchus et al [25] asserted that the context in which IPV technology was being introduced had to be considered. There should be a possibility for a patient-provider relationship in addition to IPV technology.

We also identified negative attitudes and experiences to answer questions about IPV on a tablet. For some women, participation in the tablet intervention led to further reflection on the topic. Some expressed that it was painful to be reminded about their experiences with IPV. A qualitative user-involvement study with the purpose of developing the film, which was a part of the Safe Pregnancy Study, found that women acknowledged that the film potentially could trigger painful memories [18]. This emphasizes the importance of face-to-face support from a provider as a supplement to questions about IPV on a tablet. In order not to influence the RCT outcome, health professionals in the Safe Pregnancy Study were blinded to the responses of the women, which is why all women were offered to watch a film. Thus, only women who took initiative themselves or screened positive on the routine questions on violence asked by the midwife received more follow-up if desired.

Interestingly, our study showed no real differences between ethnic Norwegian and immigrant women’s attitudes toward and experiences with the tablet intervention. This is in line with another Norwegian study that found independent of ethnicity, women said that they want to be asked and talk about IPV [16]. In line with other qualitative studies, both ethnic Norwegians and immigrant women in this study said that the questions were easy to answer, and that the topic was important [18,23]. However, some women expressed a feeling of being interrogated when answering the questions about IPV. Despite reassurance of confidentiality, there was uncertainty about their anonymity regarding the answers they gave in the questionnaire. Chang et al [23] also found that women answering questions about IPV on a tablet were uncertain who would get the information from their IPV disclosure. Previous studies indicate that anonymity is crucial for disclosing IPV [18,25]. eHealth tools aim to provide easy access to tailored health care information. However, eHealth tools might involve a risk within information security and privacy [37]. This might affect users’ willingness to share information because they reveal sensitive information [37]. This emphasizes the importance of health care professionals giving out information about the trustworthiness of eHealth technology [25].

### Limitations and Strengths

Time from participation in the tablet intervention to the interview might have caused recall bias, as several women had poor or no recollection of the film. They chose to see the questions and film again during the interview. Ideally, the interview should have been conducted shortly after the tablet intervention. The sample in this study was limited to the population in the Safe Pregnancy Study and was too small to draw conclusions about a broader population of women. It is a limitation that few immigrant women were recruited in this study. Inclusion criteria included that they had to speak Norwegian because the interviews were conducted in Norwegian. This may be the reason why this study did not find a clearer distinction between ethnic Norwegian and immigrant women. The findings in this study can be compared to other RCT studies where women’s responses to IPV technology have been examined [23-25]. It can contribute to further development of eHealth interventions for IPV screening and information about safety behaviors. The researchers who conducted the interviews were not involved in the design of the Safe Pregnancy Study. This is a strength because it might have contributed to facilitating data collection where the women could share nuanced views. The data set was read by all the authors. The interpretation and potential themes were discussed among the authors to improve the credibility of the findings.

Few studies have examined women’s attitudes and experiences with eHealth interventions to promote safety behaviors in order to reduce IPV. This study provides insights into the evaluation of complex interventions about IPV to promote safety behaviors.

### Conclusions

Women who participated in the tablet intervention had positive attitudes and experiences to answer questions about IPV and watch a film about safety behaviors on a tablet. The majority of the women in this study thought that a questionnaire on a tablet was easy to answer and they acknowledged that the MCHC was an appropriate place. They considered that the intervention might initiate and facilitate communication about IPV in antenatal care. Thus, IPV technology should be supplemented with the possibility of face-to-face communication. Women’s negative attitudes and experiences to answer questions about IPV on a tablet were mainly related to uncertainty regarding anonymity. Hence, tablet interventions to promote safety behaviors in pregnant women should be accompanied by information about women’s privacy.

## Appendix

Multimedia Appendix 1 Interview guide.

Multimedia Appendix 2 Characteristics of the study participants.

## Abbreviations

AAS: Abused Assessment Screen
IPV: intimate partner violence
MCHC: Mother and Child Health Center
RCT: randomized controlled trial
