# Supplementary material for: Pregnant Women’s Attitudes Toward and Experiences With a Tablet Intervention to Promote Safety Behaviors in a Randomized Controlled Trial: Qualitative Study
Source: JMIR Form Res. 2021 Jul 20;5(7):e28680. doi: 10.2196/28680 (PMC8335599; doi:10.2196/28680)
Supplement: Multimedia Appendix 1 [file formative_v5i7e28680_app1.docx]

Multimedia Appendix 1. Interview guide.

| **Introduction:**  Thank you for participating in this interview.  When you were pregnant and participated in the Safe Pregnancy study, you answered many questions and watched a film on a tablet. I will now carry out an interview about how you experienced this. I will use a tape recorder during our conversation. I will assure you that the interview will be handled without personal information. You have the right to know what kind of information that is registered about you and how the information is saved according to guidelines for privacy. You can withdraw your consent at any time.  Do you have any questions to the written consent?  I will now start the tape recorder  **Interview:**   1. Participation in the study   Do you remember when you participated in the Safe Pregnancy Study? Do you maybe remember how far in your pregnancy you had come? Was it in the beginning or in the end?  Can you tell me why you chose to participate in the Safe Pregnancy study?  Do you remember being asked to participate? How did you experience it?   1. Experience with the questionnaire   How did you experience answering the questionnaire?  (Do you want to see it again?)  When you now look at the questions. What do you think about them?  Some of the questions were about quality of life and intimate partner violence. Can you tell me how you experienced answering these questions?  Did you think any of the questions were difficult to answer?  What do think about answering this kind of questions on a tablet at the MCHC?   1. Experience with the film about safety behaviours   Can you tell me what you saw in the film? Do you want to see the film again?  When you now see the film again. What do you think about it?  Did you think the information in the film were relevant for you?  What do think about watching a film like this on a tablet at the MCHC?   1. Background for participating in this interview   Finally, can you tell me what made you accepted to participate in this interview? |
| --- |
