# Supplementary material for: Pregnant Women’s Attitudes Toward and Experiences With a Tablet Intervention to Promote Safety Behaviors in a Randomized Controlled Trial: Qualitative Study
Source: JMIR Form Res. 2021 Jul 20;5(7):e28680. doi: 10.2196/28680 (PMC8335599; doi:10.2196/28680)
Supplement: Multimedia Appendix 2 [file formative_v5i7e28680_app2.docx]

Multimedia Appendix 2. Characteristics of the study participants.

| **Informant** | **Control or intervention group** | **Time from participation in the tablet intervention the interview** | **Remembered the questions about violence when interviewed** | **Remembered the film when interviewed** | **Perceiving of integration. Scale 1-10/ Language**^[[1]](#footnote-1)^ |
| --- | --- | --- | --- | --- | --- |
| 1 | Control | 25 months | No | No | 8 |
| 2 | Intervention | 11 months | Yes | No | Norwegian as language at home |
| 3 | Control | 13 months | No | No | Norwegian as language at home |
| 4 | Control | 20 months | Yes | No | Norwegian as language at home |
| 5 | Control | 13 months | Yes | No | 8 |
| 6 | Intervention | 11 months | Yes | Yes | 8 |
| 7 | Intervention | 14 months | Yes | No | Norwegian as language at home |
| 8 | Intervention | 11 months | Yes | Yes | 9 |
| 9 | Intervention | 13 months | Yes | No | Norwegian as language at home |
| 10 | Intervention | 14 months | Yes | No | 5 |

1. Women who answered that they spoke Norwegian at home in in the Safe pregnancy study questionnaire were not asked question about integration [↑](#footnote-ref-1)
